# Identification of tyrosinase inhibitors from traditional Chinese medicines for the management of hyperpigmentation

**DOI:** 10.1186/s40064-015-0956-0

**Published:** 2015-04-17

**Authors:** Hsin-Chieh Tang, Yu-Chian Chen

**Affiliations:** Department of Biomedical Informatics, Asia University, Taichung, 41354 Taiwan; Human Genetic Center, Department of Medical Research, China Medical University Hospital, Taichung, 40402 Taiwan; Research Center for Chinese Medicine &Acupuncture, China Medical University, Taichung, 40402 Taiwan

**Keywords:** Tyrosinase inhibitor, Traditional Chinese medicine (TCM), Structure-based, Quantitative structure-activity relationship (QSAR), Ligand-based, Molecular dynamics (MD) simulation

## Abstract

The inhibition of tyrosinase is the most effective method to decrease melanin synthesis during the process of pigmentation. We aimed to find compounds from traditional Chinese medicines (TCM) that are more effective than the most commonly used tyrosinase inhibitor, arbutin. First, we employed homology modeling to construct a tyrosinase-modeled structure, and structure-based virtual screening to screen from 61,000 TCM compounds. We also adopted the following quantitative structure-activity relationship (QSAR) models for ligand-based validation: support vector machine, multiple linear regression, and Bayesian network. Molecular dynamics (MD) simulation was used to confirm the stability of ligand binding. We found that merresectine C might more effectively bind and inhibit the activity of tyrosinase than arbutin. This study provides useful evidence for the potential development of a novel non-toxic bleaching or whitening ingredient.

## Introduction

Hyperpigmentation, hypermelanosis, skin darkening, or tanning can be caused by ultraviolet(UV) exposure, drugs, or post-inflammatory conditions (Praetorius et al. 2013). UV radiation stimulates melanin synthesis in the epidermal melanocytes (Johnson et al. 1972). Drug-induced hyperpigmentation can be caused by many compounds including minocycline, amiodarone, oral contraceptives, and anticancer drugs (Holm and Nelson 2006; Rappersberger et al. 1989; Kim et al. 2012; Ibrahimi and Anderson 2010; Kew et al. 1977). Post-inflammatory hyperpigmentation can be caused by dermatological inflammatory diseases, or as a side effect of laser treatment (Ortonne and Bissett 2008; Fisher and James 2010). Tyrosinase function has been studied in the context of clinically significant diseases such as albinism or vitiligo (Chian and Wilgram 1967; Hertz et al. 1977; Betterle et al. 1984; Bowcock and Fernandez-Vina 2012), and tyrosinase dysfunction is responsible for these depigmentation diseases (Spritz et al. 1990; Song et al. 1994; Robert et al. 2003).

Studies on the mechanism of pigmentation and melanogenesis have been previously reported (Diffey et al. 1995; Bagnara et al. 1979). Melanocytes produce melanin, which determines differences in skin or hair color (Schallreuter et al. 1994). Melanogenesis, which is the synthesis and distribution of melanin in the epidermis, begins with the transcription of proteins required for melanin synthesis. Then, melanosomes are produced and transported to the melanocyte dendrites, and then to adjacent keratinocytes (McGuire and Moellmann 1972; Lin and Fisher 2007). Tyrosinase is a copper-binding enzyme that is produced only by melanocytic cells (Setty et al. 2008). The first biochemical survey of pigmentation was carried out on the mushroom because of its color and since then, the enzyme has been found widely distributed from bacteria to mammals (Fitzpatrick et al. 1950; Kukita and Fitzpatrick 1955; Wood and Ingraham 1965). Tyrosinase catalyzes the first two important reactions of melanin synthesis: L-tyrosinase to L-DOPA through hydroxylation and L-DOPA to dopaquinone through oxidation (Wykes et al. 1971; Pomerantz 1969; Korner and Pawelek 1982; Mirica et al. 2005). Tyrosine hydroxylase, the other DOPA-related enzyme in the nervous system, is not expressed in usual melanocytes. Tyrosinase is the central enzyme involved in eumelanin and pheomelanin synthesis via activation of melanocortin 1 receptor (MC1R), then expression of microphthalmia-associated transcription factor (MITF) (Sealy et al. 1982; Yaar 2013). Other enzymes involved in melanin synthesis include tyrosinase-related protein 1 (Trp 1) and Trp 2 (Sendoel et al. 2010). Inhibition of tyrosinase is the most effective method to decrease melanin synthesis (Bulengo-Ransby et al. 1993; Stern 2004).

By definition, true tyrosinase inhibitors are different from melanin inhibitors, which interfere with melanin formation by blocking its upstream signal transduction or downstream transportation, regardless of direct enzyme interaction. Blocking upstream signal transduction of tyrosinase includes down-regulation of MC1R activity and MITF expression. Blocking downstream transportation includes involvement in melanosomal transfer or epidermal abrasion leading to melanin loss. Tyrosinase inhibitors can chelate copper to prevent substrate binding (Bae-Harboe and Park 2012). Well-known whitening agents, kojic acid and hydroquinone, may induce adverse reactions such as skin irritation, dermatitis, depigmentation, and even cancer. Kojic acid may cause liver toxicity by increased glutathione S-transferase levels, and promote hepatocarcinogenesis (Chusiri et al. 2011; Ota et al. 2009). Hydroquinone may disturb immune response by affecting the function of endotoxin-activated neutrophils or microvascular endothelial cells (Hebeda et al. 2012; Hebeda et al. 2011). Herbs used for cosmetic whitening are widely used among Asians who practice traditional Chinese medicines (TCM). These agents are often composed of hundreds or thousands of compounds. It is difficult to distinguish which types of compounds that whiten skin effectively are safe for routine use (Ernst 2002; Chan 2011).

Searching for pure, safe, and effective ingredients that can achieve skin lightening would be beneficial. Fortunately, computational techniques have rapidly emerged in small molecular drug design (Tang and Chen 2015). TCMs used to lighten skin are often gentle, and have therapeutic advantage in some diseases. The use of a TCM database makes it possible to find new molecules that could be used as future drugs. We aimed to find potent compounds that can inhibit the activity of tyrosinase using computational simulation and the TCM Database@Taiwan (http://tcm.cmu.edu.tw/).

## Methods

### Compound database

We used the TCM Database@Taiwan (http://tcm.cmu.edu.tw/) to perform potential tyrosinase inhibitor screening. The TCM Database@Taiwan is a large database of TCM compounds and includes 61,000 small molecules. All the small molecules in the database were passed through Lipinski’s rule of five, absorption, distribution, metabolism, excretion, and toxicity (ADMET) to rule out potential toxic compounds in Discovery Studio (DS) (Chen 2011).

### Homology modeling

We acquired the human tyrosinase sequence from the Uniprot Knowledgebase (P14679). The 3D structure of tyrosinase from *Bacillus megaterium* was acquired from the Protein Data Bank (PDB ID: 3NM8). We aligned the sequence of human tyrosinase (P14679) and homologous *Bacillus megaterium* protein (3NM8) by using the “Modeler protocol" in Accelrys Discovery Studio (DS, San Diego, CA, USA). Based on the results of the sequence alignment, the percentage of identity and similarity was estimated. We used the Build Homology Models module in DS to perform homology modeling of tyrosinase. We confirmed the tyrosinase-modeled structure by Ramachandran plot with Rampage mode in DS.

### Disorder prediction

We used the PONDR-FIT protocol in the DisProt website to exclude the disordered residues of the tyrosinase 3D structure.

### Structure-based virtual screening

A docking protocol was performed with tyrosinase for all small compounds from the TCM Database@Taiwan and the control (arbutin) by LigandFit mode in DS. The protocol included hydrogen bonds (H-bond), pi bonds, and charge interactions. All docking poses between the ligand and tyrosinase were restricted by the force field of Chemistry at HARvard Molecular Mechanics (CHARMm). We also used the LIGPLOT protocol to display H-bonds and hydrophobic contact between the ligand and tyrosinase.

### Quantitative structure-activity relationship (QSAR) models

We used the support vector machine (SVM) and multiple linear regression (MLR) models and Bayesian network to predict the activities of selected TCM compounds. We obtained 24 compounds and pIC50 data of tyrosinase inhibitors from two previous studies: Lee et al. (2009) and Bandgar et al. (2012) (Lee et al. 2009; Bandgar et al. 2012). We transformed these compounds to 2D and 3D structures with ChemBioDraw software. Then, we used the Calculate Molecular Property module and Genetic Function Approximation module in DS to find and estimate the appropriate molecular descriptor for every ligand. We selected ten optimum descriptors for predicting activity. These descriptors, which constructed the SVM and MLR models, were verified by libSVM and Matlab Statistics Toolbox, respectively. We normalized the description between [−1,+1] with the SVM training model. The value of the square correlation coefficient (R^2^) was used to validate the model. We used the data from these compounds to predict the selected candidates and the control. The Bayes Net Toolbox (BNT), which is a Matlab package for Bayesian network modeling, predicted the pIC50 values. The predicted models used five-fold cross validation. We chose the highest R^2^of the SVM, MLR, and Bayesian network to be the predicted activity models.

### Molecular dynamics (MD) simulation

The trajectories of MD simulations were illustrated by the GROningen MAchine for Chemical Simulations (GROMACS) program (Stockholm, Sweden). Every ligand-tyrosinase complex passed through minimization, heating, equilibration, and production phases. We demonstrated the trajectories of root mean square deviation (RMSD), gyrate, mean square deviation (MSD), total energy, root mean square fluctuation (RMSF), and the central distance between ligand and protein. Cluster analysis, database of secondary structure assignment (DSSP), matrices of smallest distance of residues, and principal component analysis were also calculated.

### Ligand pathway

We used the CAVER software (Brno, Czech Republic) to find all possible ligand pathways while the ligand is bound with tyrosinase. The ligand pathway was also found to compute the possible tunnels inside tyrosinase to which the ligand bound. The most important parameters were set as the following description. Shell_radius, which defined the shell probe, was set at a radius of 4. Shell_depth, which specified the maximal depth of the surface region, was set at 5. Probe_radius, which identified the minimum tunnel radius, was set at 0.9 (Chovancova et al. 2012).

## Results

### Homology modeling

**Figure 1:**
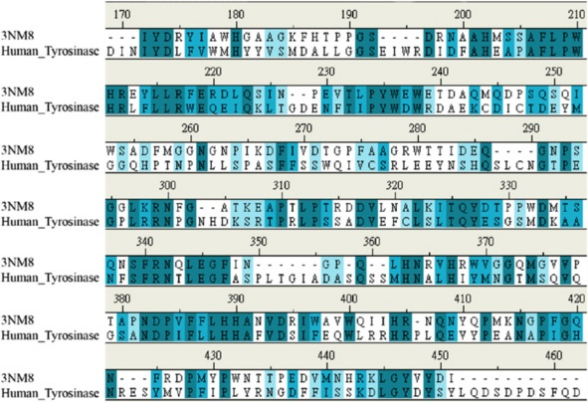
Sequence alignment between the template (3NM8) and P14679_human. The identity = 31.8% and similarity = 50.7%.

**Figure 2:**
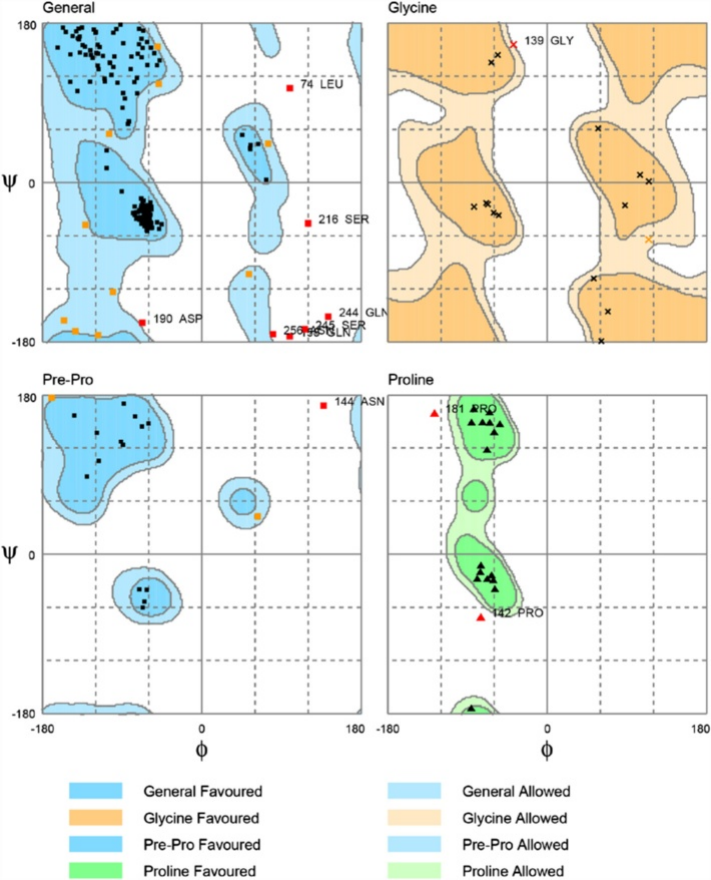
Ramachandran plot of tyrosinase-modeled structure. Number of residues in favored region (~98.0% expected) : 252 (91.3%). Number of residues in allowed region (~2.0% expected): 13 (4.7%). Number of residues in disallowed region : 11 (4.0%).

The sequence alignment between P14679_Human and the template (3NM8) had an overall identity of 31.8% and similarity was 50.7% (Figure 1). The Ramachandran plot of the tyrosinase-modeled structure demonstrates that 91.3% of residues were in the favored region, 4.7% were in the allowed region, and 4% were in the outlier region (Figure 2).

### Disorder prediction

**Figure 3:**
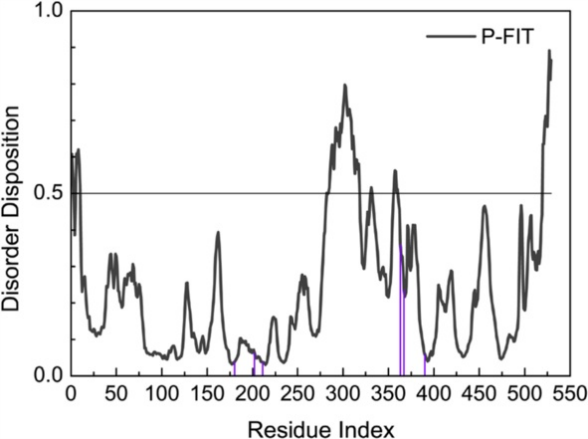
Disorder disposition of tyrosinase-modeled structure. Binding domains of main residues (illustrated in purple line) are in the non-disordered area (below the value of 0.5).

The residues of binding sites for the tyrosinase-modeled structure did not fall in the disordered area, so there was not any influence on the shape of the binding domains (Figure 3).

### Structure-based virtual screening

**Figure 4:**
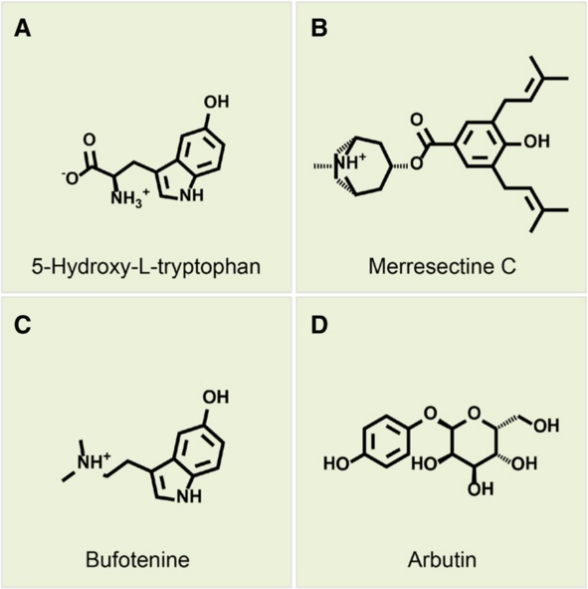
Scaffold of the top 3 TCM compounds: **(A)** 5-Hydroxy-L-tryptophan, **(B)** Merresectine C, **(C)** Bufotenine, and the control **(D)** Arbutin.

Tyrosinase catalyzes two important reactions of hydroxylation and oxidation in the presence of copper atoms. His180, His202, His211, His363, His367, and His390 were the key residues that cooperated with the copper atoms to achieve enzyme activity. The binding sites were set around the six key residues. Only 46,583 compounds could dock with the tyrosinase protein. There were 8581 compounds better than the control (arbutin) based on the Dock score. Table 1 lists the Dock score, LigScore, binding energy, H-bond quantity, pi bond quantity, and predicted activity of the top ten TCM compounds ranked by Dock score. We selected 5-hydroxy-L-tryptophan, merresectine C, and bufotenine as the candidates for further survey. Arbutin, the most commonly used tyrosinase inhibitor, was chosen as the control (Figure 4).

**Table 1:** **Top ten TCM compounds ranked by Dock score**

**Name**	**Dock score**	**LigScore**	**Binding energy**	**H-bond quantity**	**pi quantity**	**Predicted activity**		
						SVM*	MLR*	BNT*
5-Hydroxy-L-tryptophan	131.298	4.48	− 271.189	4	3	4.629	5.837	3.990
Merresectine C	124.475	4.97	− 383.479	2	2	4.694	4.194	5.742
Bufotenine	121.222	4.08	− 339.691	4	1	4.783	4.812	4.090
Physostigmine	119.468	3.55	− 355.47	1	2	4.624	5.896	5.521
Datumetine	119.025	3.66	− 355.395	2	1	4.628	5.922	5.597
Neostemonine	119.006	3.49	− 318.602	1	2	4.607	4.308	5.614
(+)-N-Methyl tryptophan methyl ester (S)	118.319	3.4	− 343.524	2	1	4.745	4.999	4.760
Stephanthrine	117.947	4.16	− 302.581	1	4	4.625	6.282	5.961
Stephenanthrine	117.947	4.15	− 302.966	1	4	4.625	6.282	5.961
Tetrahydroharmol	117.175	3.31	− 336.938	2	5	4.800	5.653	4.961
Arbutin*	60.167	4.86	− 187.707	5	0	4.871	5.710	4.331

Arbutin*: control; SVM*: support vector machine; MLR*: multiple linear regression; BNT*: bayesian network.

**Figure 5:**
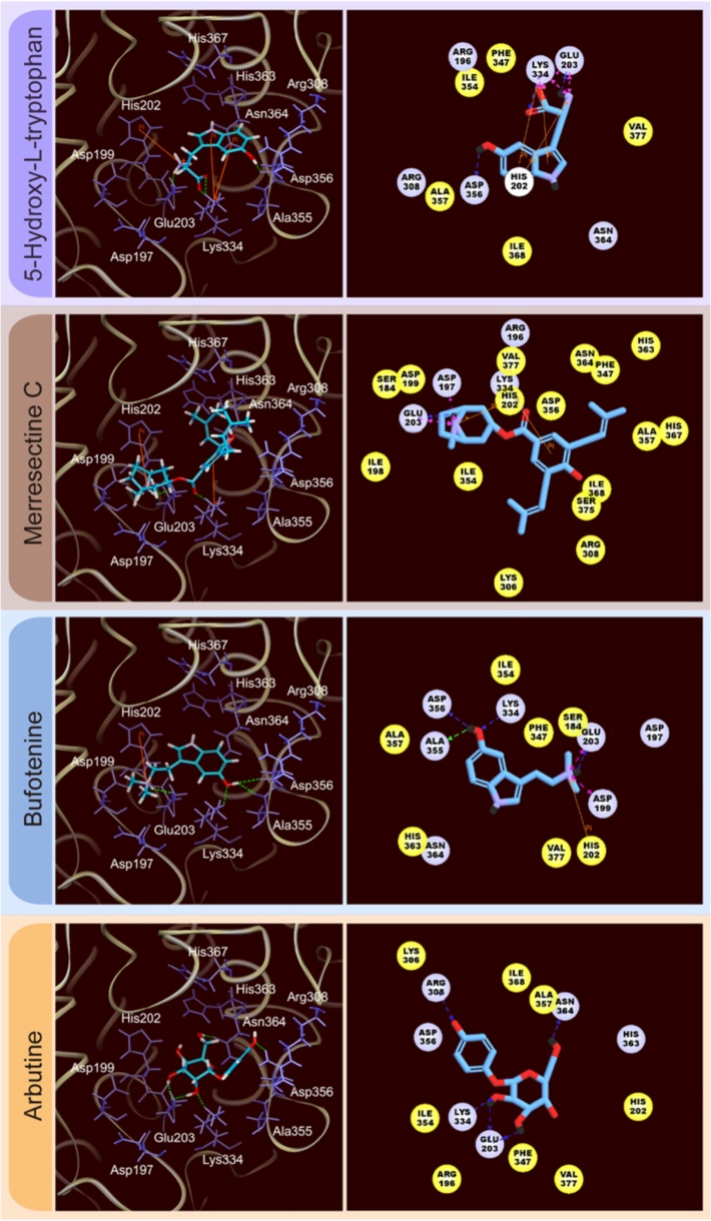
3D (left) and 2D (right) docking poses of tyrosinase. Green dashed line: H-bond with amino acids main chains; blue dashed line: H-bond with amino acids side-chains; orange line: π bond; pink dashed line: charge interaction.

**Figure 6:**
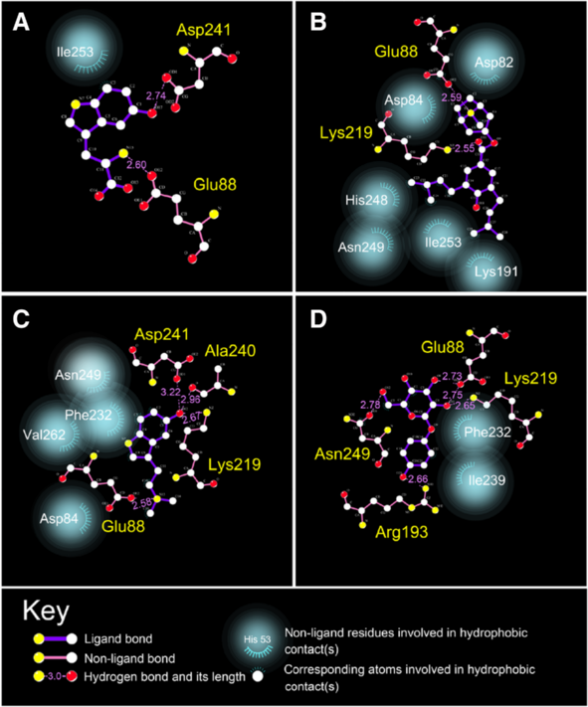
Hydrophobic contact of the ligands with tyrosinase docking poses. **(A)** 5-Hydroxy-L-tryptophan, **(B)** Merresectine C, **(C)** Bufotenine, and **(D)** Arbutin.

The binding amino acids between the ligand and tyrosinase protein were investigated. 5-hydroxy-L-tryptophan formed an H-bond with Glu203, Lys334, and Asp356. Merresectine C formed an H-bond with Glu203 and Lys334. Bufotenine formed an H-bond with Glu203, Lys334, Ala355, and Asp356. The control formed an H-bond with Glu203, Arg308, Lys334, and Asn364. The H-bond, pi bond, and charge interaction are also important binding forces between the ligand and tyrosinase. 5-hydroxy-L-tryptophan formed a pi bond with His202 and Lys334. It also had a charge interaction with Glu203 and Lys334. Merresectine C formed a pi bond with His202 and Lys334. It also had a charge interaction with Asp197 and Glu203. Bufotenine formed a pi bond with His202. It also had a charge interaction with Asp199 and Glu203 (Figure 5). Hydrophobic contact is another essential force between the ligand and tyrosinase. 5-hydroxy-L-tryptophan had hydrophobic contact with Ile253. Merresectine C had hydrophobic contact with Asp82, Asp84, Lys191, His248, Asn249, and Ile253. Bufotenine had hydrophobic contact with Asp84, Phe232, Asn249, and Val262. The control formed hydrophobic contact with Phe232 and Ile239 (Figure 6).

### Quantitative structure-activity relationship (QSAR) models

We chose the following ten optimum descriptors for constructing the ligand-based drug design models: Molecular_Solubility, Num_AromaticBonds, Num_AromaticRings, Num_AtomClasses, Dipole_X, Dipole_Y, Jurs_RASA, Strain_Energy, Shadow_XZfrac, and Shadow_YZfrac. We constructed SVM and MLR models with these descriptors. The predictive models were generated by using these descriptors:

p(IC50) = 7.636 + 0.444 × Molecular_Solubility + 0.689 × Num_AromaticBonds − 2.922 × Num_AromaticRings − 0.004 × Num_AtomClasses + 0.050 × Dipole_X − 0.107 × Dipole_Y + 0.405 × Jurs_RASA − 0.00008 × Strain_Energy − 3.092 × Shadow_XZfrac − 1.310 × Shadow_YZfrac (1)

**Figure 7:**
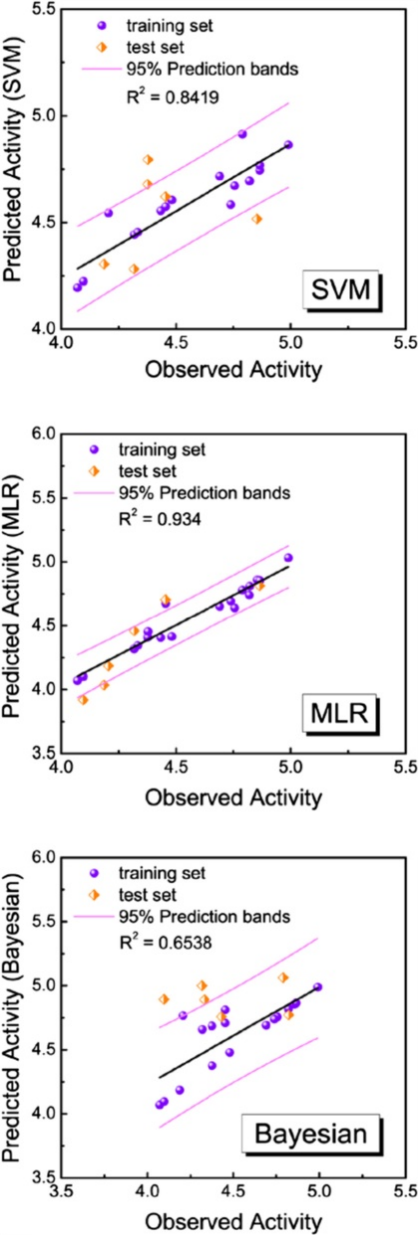
18 training sets and 6 test sets using support vector machine (SVM), multiple linear regression (MLR) and Bayesian network for predicted activity. R2 of SVM = 0.8419, MLR = 0.934 and Bayesian = 0.6538.

The 24 identified compounds were randomly divided into 18 training sets and six test sets for validation. The R^2^ values of the predicted activity for SVM, MLR, and Bayesian network were 0.8419, 0.934, and 0.6538, respectively (Figure 7).

### Molecular dynamics (MD) simulation

**Figure 8:**
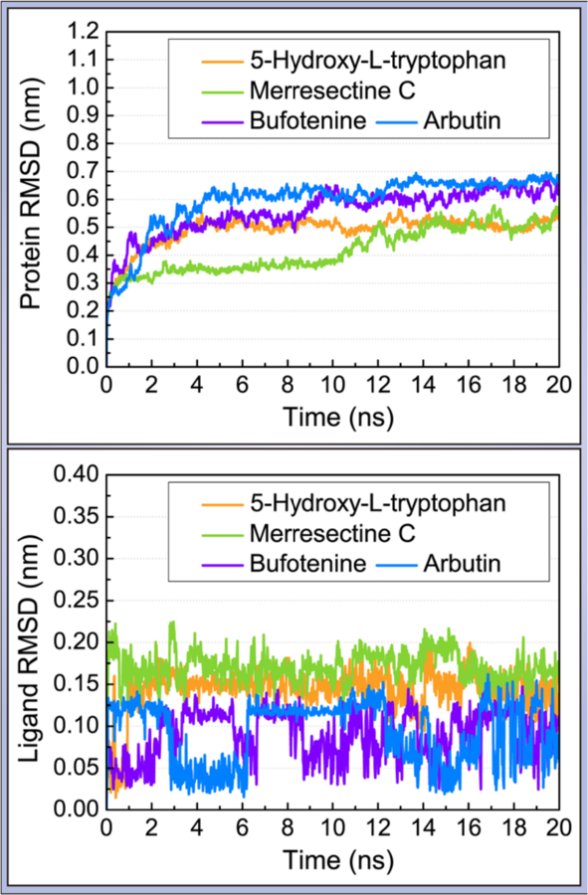
Protein and ligand root mean square deviation (RMSD).

**Figure 9:**
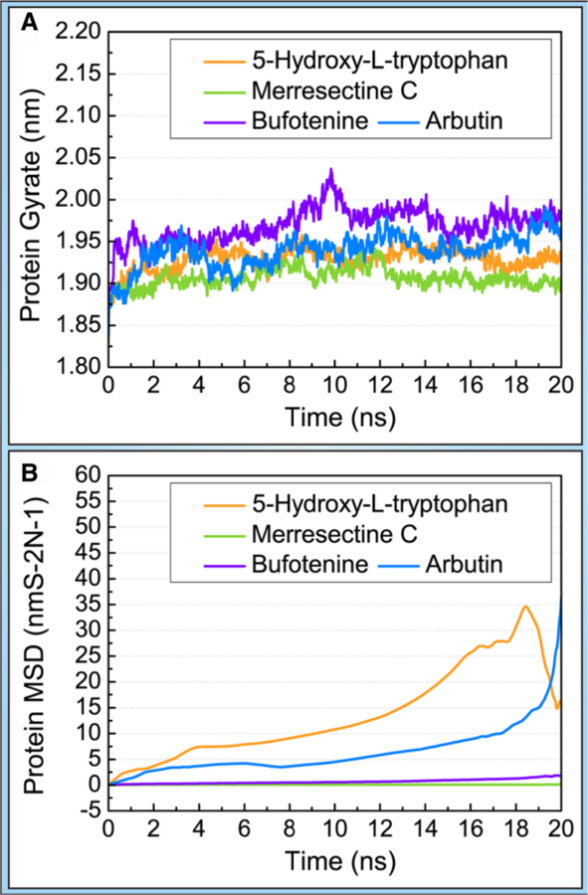
**(A)** Protein Gyrate. **(B)** Protein mean square deviation (MSD).

The trajectories of protein and ligand RMSD were drawn to compare the degree of deviation of the top three compounds and the control. The merresectine C protein-ligand complex had the lowest average protein RMSD value. The control protein-ligand complex had the largest average protein RMSD value. Conversely, merresectine C had the largest average ligand RMSD value (Figure 8). We demonstrated the trajectory of protein gyrate to investigate the average atoms’ distance to the center of every corresponding protein, which demonstrates the compact degree of every corresponding protein. The merresectine C protein-ligand complex had the lowest average protein gyrate value. The bufotenine protein-ligand complex had the largest average protein gyrate value. To calculate the deviation of each ligand-protein complex, the trajectory of protein MSD was found. The MSD trajectory of 5-hydroxy-L-tryptophan was exceeded by that of the control at the end of MD (Figure 9).

**Figure 10:**
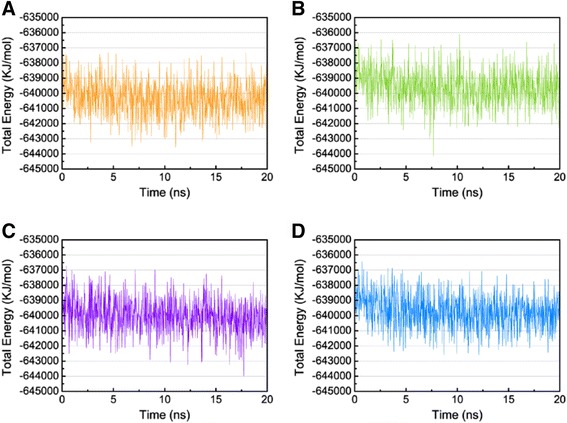
Total energy. **(A)** 5-Hydroxy-L-tryptophan, **(B)** Merresectine C, **(C)** Bufotenine, and **(D)** Arbutin.

**Figure 11:**
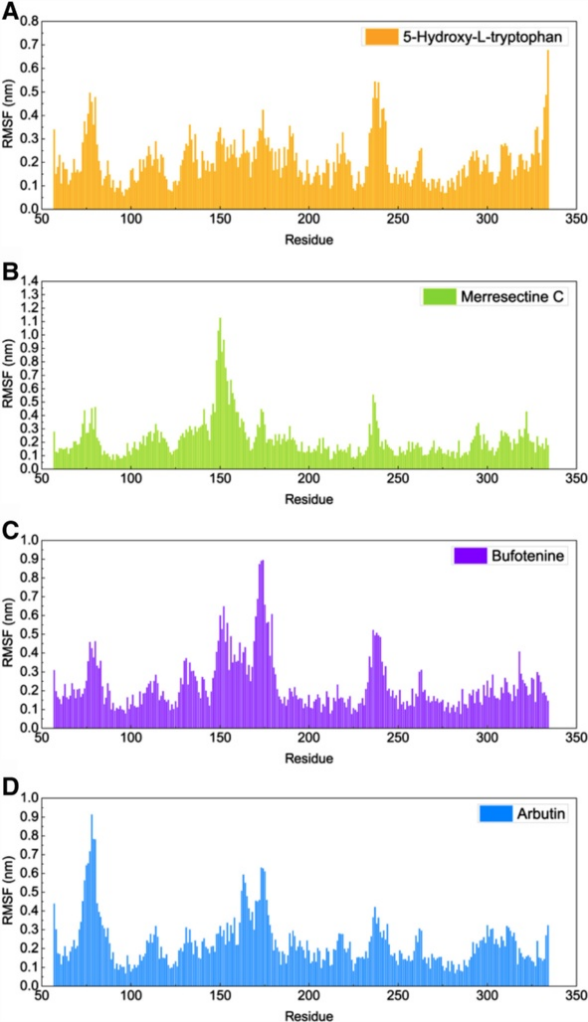
Root mean square fluctuation (RMSF). **(A)** 5-Hydroxy-L-tryptophan, **(B)** Merresectine C, **(C)** Bufotenine, and **(D)** Arbutin.

The trajectory of total energy was found to assess the stability of the ligand-protein complex. To compare the binding stability of the ligand-protein complex, we calculated the average potential and kinetic energies needed for the ligand to bind to tyrosinase (Table 2). The average total energy for 5-hydroxy-L-tryptophan, merresectine C, bufotenine and the control were −640977, −640214, −640627, and −640355 KJ/mol, respectively (Figure 10). RMSF was calculated to survey the fluctuation of every amino acid of the ligand-protein complex. The largest fluctuations of 5-hydroxy-L-tryptophan, merresectine C, bufotenine, and the control were near residues 240, 150, 175, and 80, respectively. There were no prominent influences from the key binding residues, Glu203 and Lys334 (Figure 11).

**Table 2:** **Average energy needed for the ligand-protein complex (KJ/mol)**

**Average**	**5-Hydroxy-L- tryptophan**	**Merresectine C**	**Bufotenine**	**Arbutin**
Potential energy	−784287	−783523	−783857	−783620
Kinetic energy	143310	143310	143229	143265
Total energy	−640977	−640214	−640627	−640355

**Figure 12:**
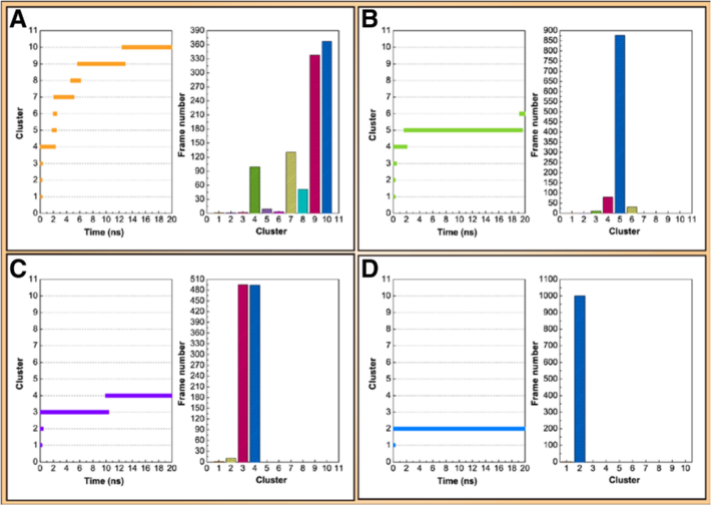
Cluster analysis. **(A)** 5-Hydroxy-L-tryptophan (−cutoff 0.14 nm), **(B)** Merresectine C (−cutoff 0.14 nm), **(C)** Bufotenine, (−cutoff 0.16 nm), and **(D)** Arbutin (−cutoff 0.153 nm).

**Figure 13:**
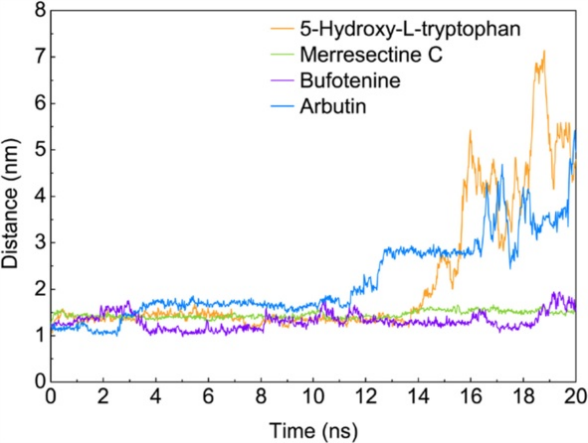
The distance of the gravity center between the ligand and tyrosinase protein.

We used cluster analysis to investigate the representative structure of the ligand-protein complex. The representative structure of the 5-hydroxy-L-tryptophan ligand-protein complex was cluster 10 from 12.5 to 20 ns. The representative structure of the merresectine C ligand-protein complex was cluster 5 from 1.5 to 19.5 ns. The representative structure of the bufotenine ligand-protein complex was cluster 3 from 0.5 to 10.5 ns. The representative structure of the control ligand-protein complex was cluster 2 from 0.5 to 20 ns (Figure 12). The distance of the gravity center between the ligand and tyrosinase was found to compare the top three candidates and the control. The control and 5-hydroxy-L-tryptophan had increased distance between the ligand and protein after 12 and 14 ns (Figure 13).

**Figure 14:**
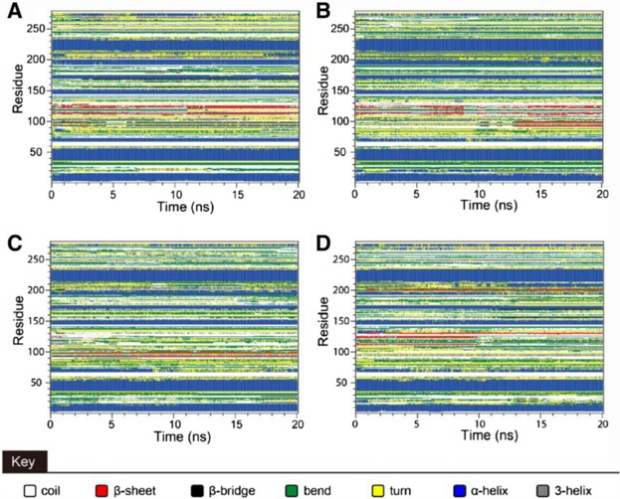
Database of secondary structure assignment (DSSP). **(A)** 5-Hydroxy-L-tryptophan, **(B)** Merresectine C, **(C)** Bufotenine, and **(D)** Arbutin.

**Figure 15:**
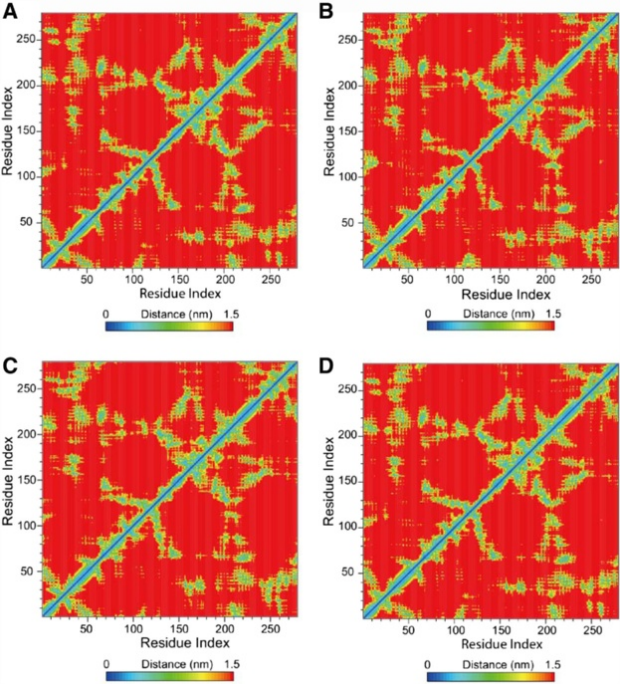
Matrices of smallest distancce of residues. **(A)** 5-Hydroxy-L-tryptophan, **(B)** Merresectine C, **(C)** Bufotenine, and **(D)** Arbutin.

Secondary structure changes were investigated to survey the structural component changes of the ligand-protein complex. There were large changes from residue 50 to 150 for the top three candidates and the control (Figure 14). Matrices of the smallest distance of residues were created to find the variation of smallest distance for any given residue. There were not any apparent differences between 5-hydroxy-L-tryptophan, merresectine C, bufotenine, or the control (Figure 15).

**Figure 16:**
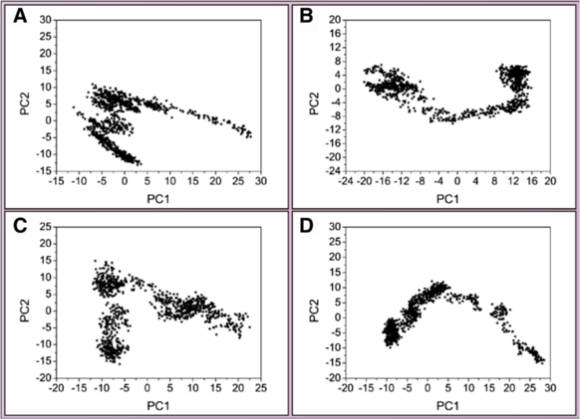
Principal component analysis (PCA). **(A)** 5-Hydroxy-L-tryptophan, **(B)** Merresectine C, **(C)** Bufotenine, and **(D)** Arbutin.

**Figure 17:**
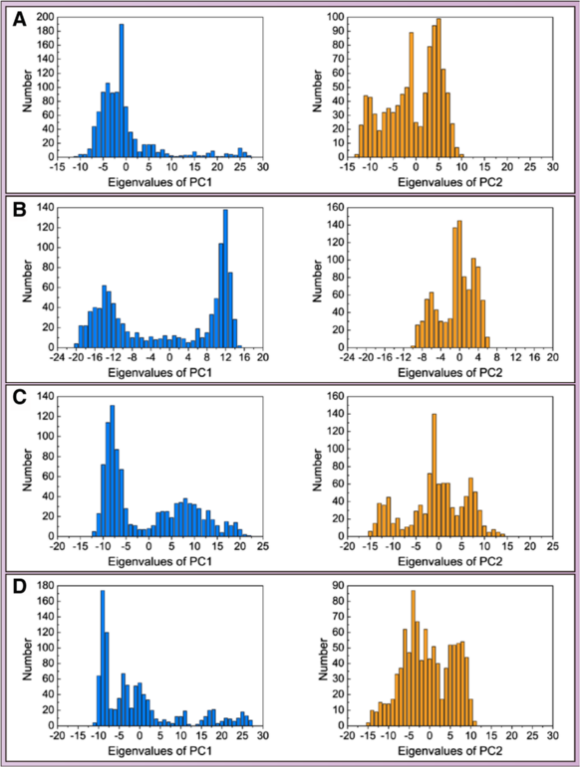
Eigenvalues of PC1 and PC2. **(A)** 5-Hydroxy-L-tryptophan, **(B)** Merresectine C, **(C)** Bufotenine, and **(D)** Arbutin.

We performed principle component analysis to find the two eigenvectors (PC1 and PC2) based on the backbone of 5-hydroxy-L-tryptophan, merresectine C, bufotenine, and the control ligand-protein complex. There were similar eigenvectors among 5-hydroxy-L-tryptophan, bufotenine, and the control (Figure 16). The eigenvalues (PC1 and PC2) were comparable with those of principle component analysis. There were similar eigenvalues of PC1 and PC2 among 5-hydroxy-L-tryptophan, bufotenine, and the control (Figure 17).

### Ligand pathway

**Figure 18:**
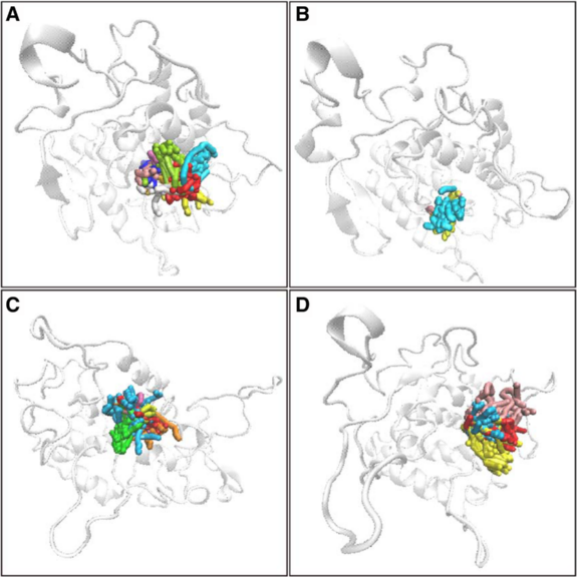
3D simulation of ligand pathway. **(A)** 5-Hydroxy-L-tryptophan (9 pathways), **(B)** Merresectine C (3 pathways), **(C)** Bufotenine(7 pathways), and **(D)** Arbutin(4 pathways).

A 3D simulation of the ligand pathway was created to estimate all possible pathways for the ligand to bind with tyrosinase. All candidates and the control had different estimated binding pathways. There were 9, 3, 7, and 4 possible pathways for 5-hydroxy-L-tryptophan, merresectine C, bufotenine, and the control, respectively (Figure 18). Aside from the binding forces of the three candidates and the control, the number and pathway of tunnels were also different.

## Discussion

### Homology modeling

We chose the human tyrosinase sequence (P14679)and the *Bacillus megaterium* (3NM8) template for homology modeling to simulate the human tyrosinase structure. 3NM8 was the most approximate crystal structure to human tyrosinase. The high percentage of identity (31.8%) and similarity (50.7%) of sequence alignment, and high percentage of residues in the favored (91.3%) and allowed (4.7%) region implied that the tyrosinase-modeled structure was reliable.

### Structure-based virtual screening

Based on the docking score, binding energy, and the quantity of important binding forces, we concluded that 5-Hydroxy-L-tryptophan, merresectine C, and bufotenine had better binding capacity than that of the control. All of the top three candidates and the control formed H-bonds with Glu203 and Lys334. Aside from the H-bonds, all of the top three candidates formed pi bonds with His202 and charge interactions with Glu203. These inhibitors occupied the original space of the copper atoms. Therefore, Glu203 and Lys334arethe key residues of the ligand-protein complex. 5-hydroxy-L-tryptophan, merresectine C, and bufotenine had more stable binding energy and binding forces than that of the control.

### Quantitative structure-activity relationship (QSAR) models

The high R^2^ values of predicted activity for SVM, MLR, and Bayesian network indicate that the predicted activity of any chosen compound is probably similar to its observed activity. The SVM values for 5-hydroxy-L-tryptophan, merresectine C, bufotenine, and the control were 4.629, 4.694, 4.783 and 4.871, respectively. The MLR values were 5.837, 4.194, 4.812, and 5.710, respectively. The BNT values were 3.990, 5.742, 4.090, and 4.331, respectively. Integrating the results of these predictive models, the MLR value of 5-hydroxy-L-tryptophan was higher than that of the control. The BNT value of merresectine C was higher than that of the control. Therefore, 5-hydroxy-L-tryptophan and merresectine C might have better biological activities than that of the control.

### Molecular dynamics (MD) simulation

Merresectine C and bufotenine had lower values for protein RMSD, MSD, and the distance of the gravity center compared with the control. The total energy result was consistent with that of protein RMSD, gyrate, and MSD. Therefore, the binding stability of merresectine C and bufotenine are probably better than that of the control, and they could bind with tyrosinase successfully and stably.

There were no similar RMSF values among 5-hydroxy-L-tryptophan, merresectine C, bufotenine, and the control. This finding was consistent with that of the cluster analysis, which showed different groups of representative structure for the top three candidates and the control. This finding implies that the dynamic condition of tyrosinase bound with each ligand was different. The principle component analysis yielded a similar finding. However, all ligands could induce changes in structure of tyrosinase. Binding of each of the top three candidates and the control resulted in large changes from residue 50 to 150 in DSSP figures. There were no apparent differences between the top three candidates and the control in the matrices of smallest residue distances. To find the individual residue or conformational changes, we conducted RMSF, cluster analysis, database of secondary structure assignment (DSSP), and the matrices of smallest distance of residues. Although the RMSF and cluster analysis patterns were different, the appearance of DSSP and the smallest distance of residues were similar. Therefore, the three candidates could induce similar changes in tyrosinase structure, similar to the control, despite the different changes in individual residues. All the ligands could therefore potentially inhibit tyrosinase activity.

**Figure 19:**
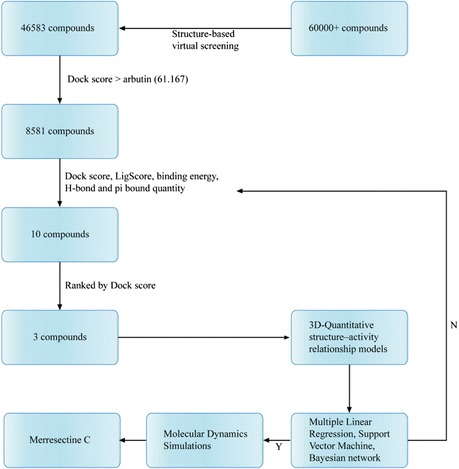
Overall filtering and verifying process.

According to the docking results, QSAR models, and MD simulation, merresectine C is the best potential lead compound for future development of a novel tyrosinase inhibitor (Figure 19).
